# pH-Sensitive Cassava Starch/Onion Peel Powder Films as Colorimetric Indicators for Minced Beef Freshness Monitoring

**DOI:** 10.3390/foods14172974

**Published:** 2025-08-26

**Authors:** Assala Torche, Toufik Chouana, Ibtissem Sanah, Fairouz Djeghim, Esma Anissa Trad Khodja, Katiba Mezreb, Redouan Elboutachfaiti, Cedric Delattre, Maria D’Elia, Luca Rastrelli

**Affiliations:** 1 Laboratory of Protection of Ecosystems in Arid and Semi-Arid Zones (Eco-Sys), Department of Biological Sciences, Faculty of Natural and Life Sciences, Kasdi Merbah University, Ouargla 30000, Algeria; torche.assala@univ-ouargla.dz; 2 Laboratoire de Génie Biologique Valorisation et Innovation des Produits Agroalimentaire, Institut ISTA-Ain M’Lila, Université Larbi Ben M’hidi Oum El-Bouaghi, Oum El-Bouaghi 04000, Algeria; sanah.ibtissem@univ-oeb.dz; 3 Laboratoire de Recherche en Sciences Alimentaires, Formulation, Innovation, Valorisation et Intelligence Art Ficielle (SAFIVIA), Institut de la Nutrition, de l’Alimentation et des Technologies Agro-Alimentaires (INATAA), Université Frères Mentouri Constantine 1, Constantine 25017, Algeria; 4 Équipe FNPAA, Laboratoire de Nutrition et Technologie Alimentaire (L.N.T.A), Institut de la Nutrition, de l’Alimentation et des Technologies Agro-Alimentaires (INATAA), Université Frères Mentouri Constantine 1, Constantine 25017, Algeria; fairouze.djeghim@umc.edu.dz; 5 Higher National School of Biotechnology Taoufik Khaznadar (ENSB), University Town, BP E6, Ali Mendjeli 25016, Algeria; tradkhodja.esma@univ-khenchela.dz; 6 UMR Transfrontalière BioEcoAgro-BiOPI, Université de Picardie Jules Verne, 80025 Amiens, France; katiba.mezreb@u-picardie.fr; 7 UMRT INRAE 1158 BioEcoAgro, BIOlogie des Plantes et Innovation (BIOPI), Avenue des Facultés, IUT d’Amiens, Université de Picardie Jules Verne, Le Bailly, 80025 Amiens, France; redouan.elboutachfaiti@u-picardie.fr; 8 Institut Pascal, Université Clermont Auvergne, Clermont Auvergne INP, CNRS, 63000 Clermont-Ferrand, France; cedric.delattre@uca.fr; 9 Department of Pharmacy, University of Salerno, Via Giovanni Paolo II, 132, Fisciano, 84084 Salerno, Italy; mdelia@unisa.it; 10 National Biodiversity Future Center (NBFC), 90133 Palermo, Italy; 11 Dipartimento di Scienze della Terra e del Mare, University of Palermo, 90133 Palermo, Italy

**Keywords:** intelligent packaging, pH-sensitive film, onion peel powder, cassava starch, meat freshness indicator, circular bioeconomy

## Abstract

pH-sensitive intelligent films offer a novel strategy for real-time monitoring of food freshness via visible color changes. This study valorizes onion peel powder (OPP), a polyphenol-rich agro-industrial by-product, by incorporating it into cassava starch-based films at three concentrations (1O, 2O, 3O). Increasing OPP content led to significantly higher total phenolic and flavonoid levels, enhancing the films’ antioxidant properties (*p* < 0.0001). While the films exhibited selective antibacterial effects, pronounced inhibition zones were observed against *Pseudomonas aeruginosa* and *Escherichia coli*, two relevant meat spoilage and pathogenic bacteria. The films displayed clear and gradual color shifts from light to dark brown across a wide pH range (1–13), confirming their suitability as pH indicators. When applied as labels in minced beef packaging stored at 4 °C, the films successfully tracked freshness over 13 days. Film color changes were strongly correlated with microbial load and pH variations, accurately flagging spoilage onset. These findings support the potential of cassava starch/OPP films as biodegradable, cost-effective intelligent packaging tools, contributing to food safety, waste reduction, and circular bioeconomy principles. The system provides a practical, non-invasive solution for meat freshness monitoring without requiring instrumentation.

## 1. Introduction

Food spoilage, particularly in protein-rich products such as meat, is a critical issue affecting food safety, economic sustainability, and consumer health. During storage, microbial metabolism leads to the accumulation of volatile nitrogenous compounds, such as ammonia and amines, which increase the pH and alter the sensory quality of the product. Traditional packaging solutions, while effective in delaying spoilage, do not provide real-time information about food freshness. In this context, intelligent packaging systems capable of indicating product quality dynamically have gained considerable attention in both academic research and industrial innovation. Among intelligent packaging systems, pH-sensitive films represent a promising strategy due to their ability to respond visibly to biochemical changes associated with microbial growth. These films typically incorporate natural pH-sensitive compounds, such as anthocyanins, into biopolymer matrices, producing materials that exhibit colorimetric shifts in response to environmental pH variations. Anthocyanins extracted from sources such as red cabbage, mulberry, and butterfly pea flower have been extensively used in this context, thanks to their vivid color changes across a wide pH range [1,2]. However, these purified pigments are often expensive, unstable to light and temperature, and require chemical extraction processes that reduce sustainability and scalability. Recent efforts have thus focused on using agro-industrial by-products as low-cost, sustainable sources of colorimetric compounds. In this study, onion peel (*Allium cepa*) powder (OPP) was selected not only for its pH-sensitive anthocyanin content and antioxidant properties, but also for its sustainable nature as an abundant agro-industrial by-product. Our formulation was optimized to balance visual responsiveness with preservation potential, aligning performance with ecological and economic considerations. Previously, we developed and characterized cassava starch-based films incorporated with onion peel powder (OPP), demonstrating their favorable antioxidant profile, mechanical integrity, and moderate antimicrobial properties, as well as visible color changes across different pH levels. The films were prepared by the solvent casting method, which involved dispersing OPP into a cassava starch solution plasticized with glycerol, followed by heating, casting onto Petri dishes, and drying under controlled conditions to obtain uniform, flexible biopolymer films [3]. Building upon those findings, the present study investigates the application of these previously developed starch/OPP films as real-time indicators of meat freshness. Specifically, we tested the films’ pH sensitivity and colorimetric response over a pH range of 1 to 13, and we applied them to monitor the spoilage of minced beef stored at 4 °C for 13 days. During storage, color variations were quantified (ΔE) and correlated with physicochemical (pH) and microbiological (aerobic plate count) changes in the meat matrix. This study aims to demonstrate the feasibility of using a biodegradable, waste-derived material as a functional freshness indicator in intelligent food packaging. Unlike prior research that relies on anthocyanin extracts or synthetic dyes [4,5], our approach emphasizes whole-powder utilization of onion peel, offering a practical and sustainable alternative for smart meat packaging applications [6,7]. Recent advances in the field demonstrate growing interest in pH-responsive smart packaging materials that incorporate natural pigments into biopolymer matrices, offering biodegradable, non-toxic alternatives for real-time freshness monitoring [8,9]. In particular, starch-based films have been recently reviewed for their use in active and intelligent packaging [10], with efforts focusing on improving mechanical and barrier functionalities while integrating bioactive compounds [11] PMC. The potential of cassava starch as a sustainable and cost-effective base—especially when blended with polymers like PVA—has also been experimentally validated in recent studies [12]. Moreover, enhancing pH sensitivity and color stability through the incorporation of bacterial nanocellulose and natural dyes such as anthocyanins has shown promising results in strengthening film performance and visual response [13,14,15].

The novelty of the present study lies in several key aspects: (i) the use of cassava starch, a cost-effective and underutilized matrix for intelligent films, particularly compared to corn or potato starch; (ii) the original combination of cassava starch with whole onion peel powder (OPP), a polyphenol-rich agro-industrial by-product, to achieve both pH-sensitivity and bioactivity; (iii) the integration of dual functionalities, active packaging (antioxidant and antimicrobial) and intelligent packaging (colorimetric freshness indicator), within a single biodegradable film; (iv) the validation of the film in a real, highly perishable food system (minced beef) under refrigerated storage, demonstrating strong correlation between visual color change, microbial growth, and pH variation. These features, together with the valorization of waste materials, position this work within a circular bioeconomy framework and highlight its potential for industrial application. In this study, onion peel powder (OPP) was selected not only for its pH-sensitive anthocyanin content and antioxidant properties, but also for its sustainable nature as an abundant agro-industrial by-product. Our formulation was optimized to balance visual responsiveness with preservation potential, aligning performance with ecological and economic considerations.

## 2. Materials and Methods

### 2.1. Materials

Cassava starch was obtained from a local supplier (Naturalim, Alger, Algeria). Onion peels (*Allium cepa*, red variety) were collected from food service waste in Oum El Bouaghi (Ain Mlila, Algeria), washed, and dried in a ventilated oven at 40 °C until a constant weight. The dried peels were then ground into a fine powder using a laboratory mill and sieved through a 45 μm mesh, as described previously [3]. Glycerol (≥99.5%) was used as a plasticizer. All other chemicals and reagents were of analytical grade and used without further purification.

Fresh minced beef was purchased from a local butcher (Salerno, Italy) and used on the same day of acquisition. Meat was divided into 50 g portions, placed in polystyrene trays, and sealed with polyethylene cling film. Samples were stored at 4 °C and monitored over time for freshness assessments using the colorimetric films.

### 2.2. Preparation of Onion Peel Powder (OPP)

Onion peels (*Allium cepa*) were collected from a local market in the Oum El Bouaghi district (Ain Mlila, Algeria). The peels were washed, cut into ~1 × 1 cm^2^ pieces, and air-dried at room temperature to a constant weight. Dried material was ground using a small-scale ball mill and sieved through a 45 μm mesh. The resulting powder (OPP) was stored in hermetically sealed bottles at room temperature until use.

### 2.3. Preparation of Indicator Films

The pH-sensitive films used in this study were prepared using the same casting method previously optimized and described in detail in our earlier work [3].

**Figure 1:**
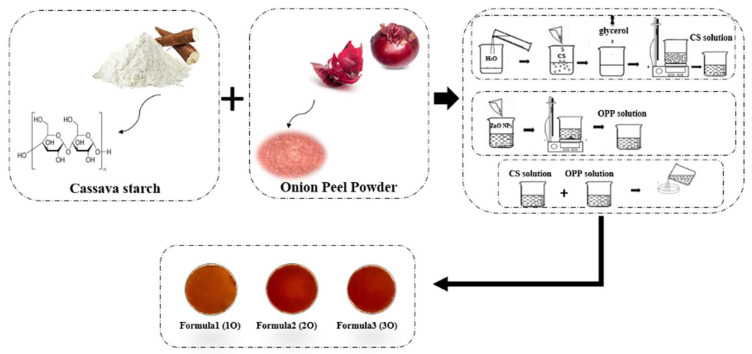
Schematic representation of the preparation of cassava starch/onion peel powder (CS/OPP) pH-sensitive films and final formulations (1O, 2O, and 3O).

The films were prepared with varying proportions of cassava starch and onion peel powder (OPP) based on statistical optimization using Minitab 19 software (Minitab Inc., State College, PA, USA) as follows: formulation 1 (1O): 72.07% cassava starch and 21.06% OPP; formulation 2 (2O): 77.28% cassava starch and 37.69% OPP; formulation 3 (3O): 84.56% cassava starch and 27.74% OPP. These values are optimization outputs and may not sum to exactly 100%, as they represent the predicted optimal proportions for targeted film properties rather than the direct final mass composition (Figure 1). These components were dissolved in 100 mL of distilled water containing 2.5% glycerol as plasticizer. The mixture (5 g total solids) was heated at 85 °C for 30 min in a shaking water bath. Then, 25 mL of each film-forming solution was cast into 10 cm diameter Petri dishes and dried at 25 ± 2 °C for 24 h. The dried films were peeled and conditioned at 50% relative humidity for 48 h before further analysis. The thickness of the dried films was measured at five random positions using a digital micrometer (accuracy ± 0.001 mm), and the mean values (±SD) were 0.18 ± 0.02 mm for formulation 1 (1O), 0.21 ± 0.02 mm for formulation 2 (2O), and 0.29 ± 0.04 mm for formulation 3 (3O).

### 2.4. Total Phenolic Content (TPC)

The total phenolic content (TPC) of the films was determined using the Folin–Ciocalteu method, with minor modifications from the procedure described by Muller et al. [16]. Briefly, 20 µL of film extract (1 mg/mL in ethanol) was mixed with 100 µL of diluted Folin–Ciocalteu reagent (1:10 *v*/*v*) and incubated for 5 min at room temperature. Then, 75 µL of sodium carbonate solution (7.5%) was added. After 2 h of incubation in the dark at room temperature, absorbance was measured at 765 nm using a microplate reader. TPC was calculated using a gallic acid standard curve and expressed as mg gallic acid equivalents per gram of film (mg GAE/g film).

### 2.5. Total Flavonoid Content (TFC)

The total flavonoid content (TFC) was measured according to the aluminum chloride colorimetric method, as described previously [17]. A 50 µL aliquot of film extract (1 mg/mL) was mixed with 130 µL of methanol, 10 µL of 1 M potassium acetate, and 10 µL of 10% aluminum nitrate. The mixture was incubated at room temperature for 40 min, and the absorbance was recorded at 430 nm. TFC was calculated using a quercetin standard curve and expressed as mg quercetin equivalents per gram of film (mg QE/g film).

### 2.6. Antioxidant Activity (DPPH and ABTS Assays)

The antioxidant activity of the films was evaluated using 2,2-diphenyl-1-picrylhydrazyl (DPPH) and 2,2′-azino-bis-(3-ethylbenzothiazoline-6-sulfonic) acid (ABTS) radical scavenging assays, following the method of Dou et al. [18] with minor modifications. Film samples (4 mg) were dissolved in a DMSO–ethanol mixture (10% *v*/*v*).

For the DPPH assay, 160 µL of 0.1 mM DPPH solution was mixed with 40 µL of the film extract and incubated in the dark at room temperature for 30 min. Absorbance was measured at 517 nm. Radical scavenging activity was calculated using the following equation:

(1) $$DPPH(\%)=\frac{A0-A1}{A0}\times 100$$

where A0 is the absorbance of the control and A1 is the absorbance of the sample.

For the ABTS assay, a 40 µL aliquot of the same extract was mixed with 160 µL of ABTS working solution and incubated for 15 min in the dark. Absorbance was recorded at 734 nm, and the ABTS scavenging activity was calculated using the same formula.

### 2.7. Release Rate of Antioxidants

The release of antioxidants from the films was assessed according to Pirsa et al. [19]. Film samples (2 × 2 cm) were immersed in 20 mL of deionized water for 6 h, with gentle stirring every 20 min. After incubation, the solution was filtered and analyzed spectrophotometrically in the range 200–800 nm. The percentage release was calculated using Equation (2):

(2) $$RELEASE\;(\%)=\frac{A0-A1}{A0}\times 100$$

where A0 is the absorbance of the original diluted onion peel extract used to prepare the film and A1 is the absorbance of the released fraction.

### 2.8. Antibacterial Activity

The antibacterial activity of the OPP-incorporated films was evaluated using the agar disk diffusion method [20]. Four bacterial strains were selected: two Gram-negative strains (*Escherichia coli* CIFA 25922 and *Pseudomonas aeruginosa* ATCC 27853) and two Gram-positive strains (*Staphylococcus aureus* ATCC 25923 and *Bacillus cereus* ATCC 11778).

Sterile disks of the film samples (15 mm diameter) were placed onto Mueller–Hinton agar plates previously inoculated with 10^6^ CFU/mL of each bacterial suspension. The plates were incubated at 37 °C for 24 h, and the diameter of the inhibition zones was measured in millimeters. Antibacterial efficacy was determined based on the presence and size of clear inhibition zones surrounding the film disks.

### 2.9. pH-Sensing Ability of OPP and Starch–OPP Films

The pH-sensing ability of the films was evaluated by immersing OPP-loaded cassava starch films (4 mg) in 10 mL of buffer solutions with pH values ranging from 1.0 to 13.0. The color response of each solution was visually observed and photographed.

Following the method of Luchese and Sperotto [21], film samples were also directly immersed in the same buffer solutions for 10 min, then removed and dried prior to colorimetric analysis. Color parameters (L*, a*, b*) were measured using a portable colorimeter, and the total color difference (ΔE*) was calculated according to the CIE L*a*b* (CIELAB) system using the following Equation (3):

ΔE = [(L* − L_0_)^2^ + (a* − a_0_)^2^ + (b* − b_0_)^2^] ½ (3)

where L_0_ = 54.433, a_0_ = 31.6, and b_0_ = 43.7333.

### 2.10. Performance of the Real-Time Monitoring of Meat Freshness

#### 2.10.1. Packaging of Minced Beef

Fresh minced beef was obtained from a local supermarket and aseptically portioned into buckle-type Petri dishes (~90 mm diameter), each containing approximately 30 g of meat. Intelligent film labels (cut to match the lid size) were affixed to the inner side of the dish covers, ensuring no direct contact with the meat. All samples were stored at 4 °C under refrigeration, and observations were carried out on days 0, 3, 5, 7, 9, 11, and 13. Color changes in the films were visually monitored and photographed and were further correlated with microbiological and physicochemical parameters of the meat.

#### 2.10.2. Microbiological Analysis

To evaluate microbial spoilage, 10 g of each meat sample was aseptically transferred to sterile bags containing 90 mL of sterile peptone water and homogenized using a Stomacher (HG 400 Pro, Wiggens, Wuppertal, Germany). Serial decimal dilutions were prepared and plated onto Plate Count Agar (PCA). The plates were incubated at 30 °C for 48 h in accordance with ISO 4833-1:2013 [22]. Aerobic plate counts (APCs) were expressed as log_10_ CFU/g. Measurements were performed at each time point (0, 3, 5, 7, 9, 11, and 13 days). The total viable count was calculated using the following Equation (4):

(4) $$N=\frac{\Sigma C}{V\;(n1+0.1\times n2)\;D}$$

where

- *N* = number of colonies per mL or g of product;
- *ΣC*\Sigma *ΣC* = total number of colonies counted on both dishes;
- V = volume of inoculum used (mL);
- n1 = number of plates at the first dilution;
- n2 = number of plates at the second dilution;
- D = dilution factor corresponding to the first dilution.

#### 2.10.3. pH Measurement

The pH of the meat was determined following the method of Smaoui et al. [23]. Briefly, 10 g of meat was homogenized with 100 mL of distilled water (1:10 *w*/*v* ratio), and the mixture was filtered. The pH of the filtrate was measured using a calibrated pH meter (Crison Basic 20, Crison Instruments, Alella, Spain). Measurements were conducted in triplicate on each storage day.

#### 2.10.4. Colorimetric Analysis of Indicator Films

The colorimetric response of the intelligent films was quantitatively assessed using a colorimeter in the CIELAB color space. The parameters L* (lightness), a* (red–green), and b* (yellow–blue) were recorded at each time point (0, 3, 5, 7, 9, 11, and 13 days). Each measurement was performed in triplicate. The total color difference (ΔE*) was calculated according to Equation (3).

### 2.11. Statistical Analysis

All experiments were conducted in triplicate, and results were expressed as mean ± standard deviation. One-way analysis of variance (ANOVA) was performed to evaluate significant differences among the experimental groups. Tukey’s multiple comparison test was applied at a significance level of *p* < 0.05. All statistical analyses were conducted using XLSTAT 2019 software (Addinsoft, Paris, France).

## 3. Results

### 3.1. Total Phenolic and Flavonoid Content

The total phenolic content (TPC) of the OPP films is shown in Table 1. Since cassava starch is naturally devoid of bioactive compounds, the observed significant increase (*p* < 0.05) in TPC with higher levels of OPP incorporation is directly attributable to the phenolic constituents of the onion peel powder.

Among all formulations the 2O film exhibited the highest TPC, while the 1O film displayed the lowest value (*p* < 0.0001), as reported in Table 1. This positive trend confirms the contribution of OPP as a rich source of phenolic compounds. Our results are in line with studies by Kumar et al. [17] and Medeiros Silva et al. [24], demonstrating that plant-based additives enhance the antioxidant profile of biopolymer films.

A similar pattern was observed for total flavonoid content (TFC), also reported in Table 1, with significant enhancement (*p* < 0.0001) upon OPP incorporation. The increase in TPC and TFC reflects the presence of phytochemicals such as quercetin and anthocyanins abundant in onion peel [25], supporting findings from Akcan et al. [26] on flavonoid-rich films.

### 3.2. Antioxidant Activity

The antioxidant activities of the films, measured via DPPH and ABTS radical scavenging assays, are shown in Table 1. Cassava starch films alone displayed minimal activity (DPPH: 12.95%; ABTS: 42.94%), whereas OPP-enriched films, particularly the 2O formulation, exhibited significantly higher activity (DPPH: 87.50%; ABTS: 90.60%; *p* < 0.0001).

The enhanced activity correlates with the increasing TPC of the films and reflects the potent antioxidant capacity of onion peel [25]. This confirms the results of [24], which demonstrated that incorporating onion waste into biodegradable matrices increases antioxidant effectiveness.

Interestingly, the ABTS activity exceeded DPPH activity, likely due to the higher hydrophilicity of compounds such as quercetin and anthocyanins, which interact more readily with ABTS radicals. This disparity has also been noted in prior studies [26,27], reinforcing the role of phenolic-rich matrices in active packaging. The superior antioxidant activity of the 2O formulation may be explained by an optimal balance between OPP loading and matrix dispersion, where the phenolic compounds are present in sufficient concentration and remain well-distributed, maximizing radical scavenging efficiency; in contrast, lower OPP content (1O) reduces bioactive availability, while excessive OPP (3O) can promote particle agglomeration and limit active site accessibility.

### 3.3. Release of Antioxidant Compounds

**Figure 2:**
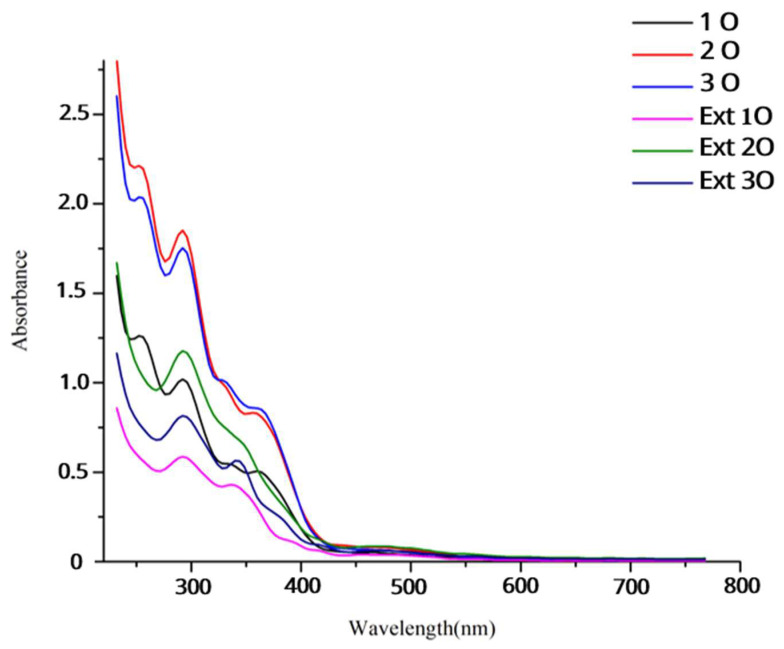
UV–vis spectra in the range of 200–800 nm for OPP films aqueous extract (1O, 2O, 3O) and OPP solution (ext 1O, ext 2O, ext 3O).

The UV–vis spectra (200–800 nm) of OPP films and their extracts (Figure 2) confirm the presence of characteristic phenolic compounds. Peaks at 260 and 375 nm are consistent with quercetin, while a lower-intensity band at 530 nm corresponds to anthocyanins—typical pigments in red onion peel.

As shown in Table 2, the release of antioxidants from the films into aqueous medium over 6 h followed a positive trend with OPP concentration, with formula 3O releasing the highest amount. The dominant 375 nm peak was used as a marker for quercetin migration. These findings align with Thi Nguyen et al. [28], suggesting aqueous compatibility of phenolic compounds for film-based release systems.

The increase in the height of the peaks at ~260 nm and ~530 nm over time reflects a higher concentration of released compounds, rather than the formation of new molecules. The 260 nm peak is primarily associated with quercetin and its derivatives, while the 530 nm peak corresponds to anthocyanins, which are responsible for the red/violet coloration of onion peel. As the films interact with the aqueous medium, these water-soluble phenolic compounds are progressively released, contributing to both antioxidant activity and the pH-sensitive colorimetric response. Given that the spoilage of minced beef is mainly driven by biochemical and microbial changes in the aqueous phase, the release of water-soluble compounds was the primary focus of this study, while potential lipid-soluble fraction release was not considered to have a significant impact on the indicator function.

### 3.4. Antimicrobial Properties

Representative images of inhibition zones for each formulation and microorganism are provided in Supplementary Material (Figure S1), while quantitative measurements are summarized in Table 3. While the control starch film exhibited no inhibition, OPP films displayed strain-specific antimicrobial activity, particularly against *Pseudomonas aeruginosa* (formulations 1O and 2O) and *Escherichia coli* (1O). These results are in agreement with earlier studies [29], highlighting the antimicrobial efficacy of onion peel extracts.

Interestingly, a lower concentration (1O) was more effective against *E. coli*, indicating that inhibition may not be strictly dose-dependent and could reflect a balance of phytochemical composition and microbial susceptibility.

The antimicrobial activity is primarily attributed to polyphenols, which disrupt microbial membranes and metabolic pathways [30,31]. The observed antimicrobial activity of the OPP-based films is primarily attributed to phenolic compounds such as quercetin and anthocyanins, which are abundant in onion peel powder (OPP). The mechanism of action of these polyphenols is multifactorial. They can disrupt bacterial cell membrane integrity, leading to leakage of intracellular components and destabilization of membrane potential. Additionally, they may bind to and inhibit key microbial enzymes, interfering with energy metabolism and nucleic acid synthesis. Notably, the antimicrobial effect was not strictly dose-dependent; for example, the 1O formulation, with a lower OPP content, inhibited *E. coli*, whereas higher OPP concentrations in 2O and 3O did not. This suggests that an optimal balance between phytochemical composition and bacterial susceptibility is crucial for effective inhibition, and that excessive pigment or particle aggregation in higher OPP formulations might reduce the availability of active sites. The selective inhibition observed for *P. aeruginosa* and *E. coli* can be attributed to their Gram-negative cell wall structure, which includes an outer membrane rich in lipopolysaccharides (LPSs). Phenolic compounds in OPP are known to interact with and disrupt this outer membrane, increasing permeability and causing leakage of intracellular components. In contrast, the thick peptidoglycan layer of Gram-positive bacteria may act as a more effective barrier against these compounds, explaining the absence of inhibition zones in the tested Gram-positive strains [32].

### 3.5. pH-Sensitive Color Change

**Figure 3:**
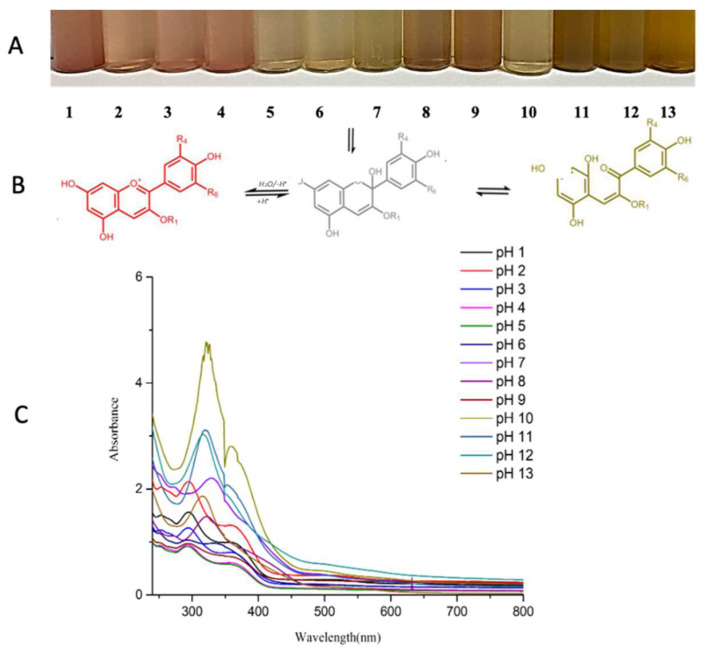
(**A**) Visual color variations in onion peel extract (OPP) solutions across a pH range from 1 to 13. (**B**) Proposed molecular structure transformations of flavonoids under acidic and alkaline conditions. (**C**) UV–visible absorption spectra of OPP in different buffer solutions (pH 1 to 13), highlighting pH-dependent optical behavior relevant for colorimetric freshness indicators.

The colorimetric and UV–vis responses of OPP to varying pH conditions are shown in Figure 3A,B. OPP solutions exhibited color shifts from reddish-pink (pH 1–3) to pale pink (neutral) and yellow (alkaline). These changes are associated with structural transitions of anthocyanins [33].

Spectral peaks at 240–280 nm and 300–380 nm represent the benzoyl and cinnamoyl systems of flavonoids, consistent with prior reports [34]. The ΔE values (color difference) recorded in Table 4 confirm that the films are visually responsive to pH shifts, supporting their utility in smart packaging.

The maximum ΔE value of 47.977 at pH 13 implies a strong visual cue even for untrained consumers [35], highlighting the film’s efficacy as a pH indicator. The ΔE values did not follow a perfectly linear increase across the pH range, but instead showed pronounced differences under extreme acidic (pH 1) and alkaline (pH 12–13) conditions, with intermediate values at neutral pH. This non-linear trend reflects structural transformations of onion peel anthocyanins, which exhibit distinct chromatic characteristics in acidic versus alkaline environments, confirming the films’ high responsiveness to pH changes.

### 3.6. Real-Time Monitoring of Meat Freshness

**Figure 4:**
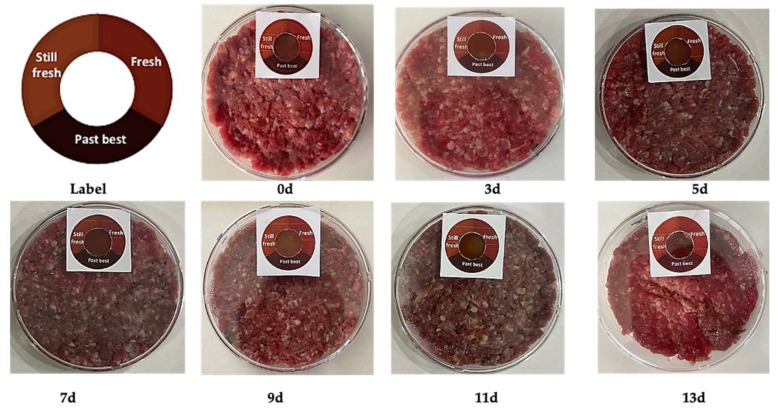
Visual reference chart of the colorimetric label indicating freshness stages of minced beef: “Fresh,” “Still fresh,” and “Past best” packages stored at 4 °C over 13 days (0d, 3d, 5d, 7d, 9d, 11d, 13d).

Colorimetric labels applied to minced beef packages stored at 4 °C revealed distinct changes (Figure 4) over 13 days. Initial microbial counts (5.4 log CFU/g) increased steadily, surpassing the quality threshold of 6.0 log CFU/g by day 9 and peaking at 8.80 log CFU/g (Table 5).

**Figure 5:**
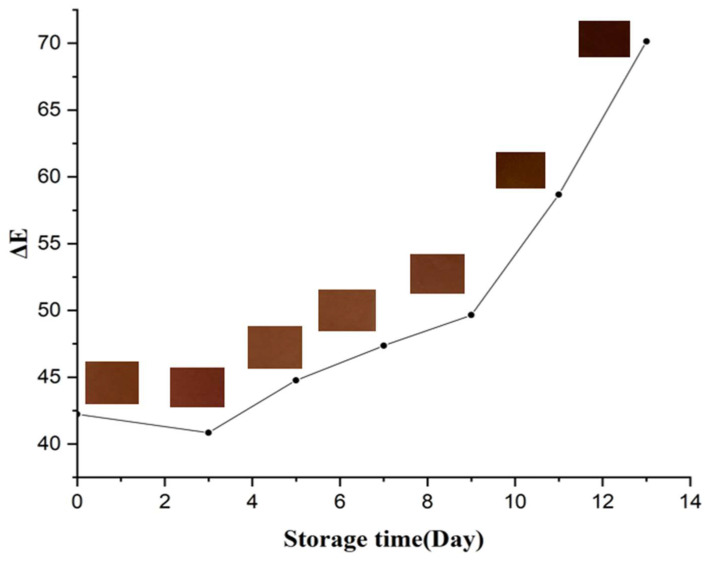
Correlation between ΔE values and storage time (days) for minced beef meat stored at 4 °C. The color swatches represent visual changes in the pH-sensitive film label over time, showing a progressive browning trend corresponding to meat spoilage.

In parallel, meat pH increased significantly from 6.043 to 9.57 (*p* < 0.0001), confirming spoilage due to protein degradation and microbial by-products [36,37]. A robust correlation was observed between microbial growth, pH increase, and the ΔE values of the OPP films (Figure 5), which shifted from brown to dark brown with advancing spoilage. These results confirm the film’s ability to visually and quantitatively monitor food freshness. Similar performance was reported by Elhadef et al. [20] using carboxymethyl cellulose/date pit anthocyanin films. It should be noted that the photographic representation of the films, especially when placed directly on minced beef, may underestimate the perceived color difference due to lighting conditions, shooting angle, and background interference from the meat surface. In practical applications, when the film is inspected separately under neutral lighting, the pH-induced color change is clearly visible, in agreement with the ΔE values that exceed the commonly accepted perceptibility threshold (ΔE > 3).

## 4. Discussion

The current study reinforces the suitability of onion peel powder (OPP) as a functional additive for intelligent packaging applications, building upon our previous investigations on OPP’s phytochemical composition and bioactivity [38] and its use as a reinforcing agent in biodegradable cassava starch-based films [3]. In the latter work, the synergistic effect of cassava starch and OPP improved mechanical strength and biodegradability, while also introducing antioxidant properties due to the high flavonoid content of onion peels. These findings provided the conceptual and technological basis for the present study, in which OPP was employed not only as a structural component but also as a natural pH-sensitive dye. The resulting films were successfully applied as colorimetric indicators to monitor the freshness of minced beef during refrigerated storage, thus extending the application potential of OPP-based films beyond structural and biodegradable matrices to active and intelligent packaging systems.

### 4.1. Food Safety and Shelf-Life Extension

The intelligent label developed in this study demonstrated a dynamic and reliable response to two of the most critical indicators of meat spoilage: microbial growth and pH increase. Notably, the films showed a strong correlation between ΔE values, microbial load, and pH, with a visible color transition that matched the critical spoilage threshold (6 log CFU/g). This visible change, from light brown to dark brown, occurred between day 7 and day 9 of storage and provided an intuitive and non-invasive tool for monitoring freshness in real time. The results are consistent with those of Zhao et al. [39], who reported the pH-sensitivity and freshness-monitoring capacity of anthocyanin-based films in various food matrices, including fish and shrimp. However, while anthocyanins require careful stabilization due to their thermal and oxidative lability, our work demonstrates that onion peel polyphenols maintain color stability and responsiveness under refrigerated conditions, offering a more robust and cost-effective alternative. Moreover, unlike many anthocyanin-based films that rely on synthetic polymer supports (e.g., PVA or chitosan blends) to enhance barrier properties, the present OPP films are fully biodegradable and derived from food waste, associating them with circular economy principles.

This improvement in pH-responsiveness and the ability to detect spoilage onset enables better shelf-life management, inventory control, and waste reduction. Such early and clear detection not only enhances food safety, by reducing the risk of consuming contaminated meat, but also offers commercial benefits in retail environments where visual indicators can build consumer trust. The simplicity, low cost, and eco-compatibility of the developed films further supports their scalability in the meat industry and beyond.

### 4.2. Advantages of OPP-Based Colorimetric Films

The OPP-based colorimetric films developed in this work present several notable advantages for intelligent packaging applications. First, they valorize onion peel powder (OPP), an agro-industrial by-product, thereby contributing to circular economy strategies and reducing environmental impact. Second, they combine dual functionality, acting as intelligent packaging by providing a visible freshness indicator through pH-dependent color change and as active packaging due to the antioxidant and antimicrobial properties conferred by OPP’s phenolic compounds (notably quercetin), which may help slow spoilage. Third, the films are produced using cassava starch, a biodegradable, renewable, and widely available polymer, offering an eco-friendly alternative to petroleum-based plastics. Finally, the use of low-cost raw materials such as cassava starch and onion peel enhances the potential cost-effectiveness and scalability of the system. These combined features position the developed films as a multifunctional, sustainable, and commercially viable packaging solution for highly perishable foods such as minced beef.

### 4.3. Circular Bioeconomy and Valorization of Agro-Waste

This work exemplifies a circular bioeconomy model, transforming onion peel, an abundant agro-industrial waste, into a high-value functional packaging component. Onion peels are rich in polyphenols and flavonoids, compounds that retain antioxidant and antimicrobial properties even after incorporation into the cassava starch matrix. Such waste-derived ingredients not only enhance the functional profile of biodegradable films but also add environmental and economic value, reducing landfill waste and promoting resource efficiency.

By replacing synthetic additives and petrochemical-based materials, OPP films contribute to reducing the environmental footprint of food packaging. The biodegradability of the cassava starch base further supports a low-impact end-of-life profile, aligning with global sustainability goals.

### 4.4. Industrial Relevance and Innovation

The intelligent, pH-sensitive label developed in this work combines active and intelligent packaging functions in a single biodegradable system. It is fully compostable, non-toxic, and avoids direct contact with meat, ensuring food safety while enabling real-time freshness detection without instrumentation. The visible color changes are easily interpreted by consumers, facilitating practical adoption in retail environments.

From a manufacturing standpoint, the fabrication process of simple casting and drying is cost-effective, scalable, and compatible with current packaging lines. This makes the system attractive for food processors, retailers, and smart-packaging developers seeking sustainable solutions to extend shelf-life and improve quality monitoring. By merging waste valorization with intelligent food monitoring, this innovation bridges sustainability goals with market-ready applicability.

### 4.5. Future Perspectives

Further work will be needed to test these intelligent labels on other high-protein perishable products such as fish, poultry, and ready-to-eat meats, as well as to optimize label printing, adhesion, and integration in industrial settings. Moreover, expanding the colorimetric scale and integrating QR-based digital tracking could further enhance functionality.

## 5. Conclusions

This study demonstrates the successful development of cassava starch-based intelligent films incorporating onion peel powder (OPP), an agro-industrial by-product rich in bioactive compounds. The resulting biopolymer films exhibited significant antioxidant activity, moderate antimicrobial effects, and strong pH-sensitivity, making them highly suitable for smart packaging applications.

Notably, OPP films were effectively employed as real-time visual indicators of beef meat freshness during 13 days of refrigerated storage. Color changes in the indicator films closely mirrored the spoilage process, correlating with increases in microbial load and pH levels. These findings confirm the feasibility of using OPP-based films as non-invasive freshness sensors in meat packaging, in agreement with the core principles of intelligent packaging systems that monitor product quality during storage and distribution.

Beyond their functional performance, the use of biodegradable and waste-derived materials positions these films within a circular bioeconomy framework, contributing to both food safety and sustainability. This work provides a strong foundation for the industrial application of such labels in perishable protein-based foods, offering a low-cost, environmentally friendly, and consumer-friendly tool for enhancing food quality management and reducing waste. Future studies will include the measurement of total volatile basic nitrogen (TVB-N) alongside pH and microbial counts, to provide a more comprehensive assessment of the films’ preservation effect on minced beef.

## Figures and Tables

**Table 1:** Total phenolic content (TPC), total flavonoid content (TFC), and radical scavenging activity (DPPH and ABTS assays) of cassava starch/OPP films.

Films	DPPH (%)	ABTS (%)	TPC (µg/mL)	TFC (µg/mL)
Control	12.95 ± 0.03 ^b^	42.94 ± 0.15 ^c^	63.71 ± 1.93 ^b^	15.97 ± 3.24 ^c^
Formula 1 (1O)	23.28 ± 3.31 ^b^	88.38 ± 0.51 ^b^	97.82 ± 2.22 ^a^	29.65 ± 3.83 ^b c^
Formula 2 (2O)	87.50 ± 8.55 ^a^	90.60 ± 0.60 ^a^	109.39 ± 2.07 ^a^	88.54 ± 3.09 ^a^
Formula 3 (3O)	66.62 ± 13.76 ^a^	89.03 ± 0.80 ^b^	102.33 ± 9.85 ^a^	43.82 ± 1.62 ^b^
*p*-Value	*p* < 0.0001	*p* < 0.0001	*p* < 0.05	*p* < 0.0001

Data are mean values ± standard deviations. Values in each column with different letters (a–c) are significantly different (*p* < 0.05).

**Table 2:** Release rate of antioxidants from the OPP films into the aqueous solution within 6 h.

Films	Antioxidant Release (%)
Formula 1 (1O)	70.10 ± 0.005 ^c^
Formula 2 (2O)	95.03 ± 0.025 ^a^
Formula 3 (3O)	83.31 ± 0.015 ^b^
*p*-value	*p* < 0.0001

Data are mean values ± standard deviations. Values in each column with different letters (a–c) are significantly different (*p* < 0.05).

**Table 3:** Inhibition zone diameters for OPP film formulations against selected pathogens.

Films	*Bacillus cereus*	*Pseudomonas aeruginosa*	*Escherichia coli*	*Staphylococcus aureus*
Control	0.0 ± 0.0 ^c^	0.0 ± 0.0 ^c^	0.0 ± 0.0 ^b^	0.0 ± 0.0 ^b^
Formula 1 (1O)	0.0 ± 0.0 ^c^	8.06 ± 0.04 ^c^	7.22 ± 0.02 ^a^	0.0 ± 0.0 ^b^
Formula 2 (2O)	0.0 ± 0.0 ^c^	11.04 ± 0.03 ^b^	0.0 ± 0.0 ^b^	0.0 ± 0.0 ^b^
Formula 3 (3O)	0.0 ± 0.0 ^c^	0.0 ± 0.0 ^a^	0.0 ± 0.0 ^b^	0.0 ± 0.0 ^b^
*p*-Value	*p* > 0.05	*p* < 0.0001	*p* < 0.0001	*p* > 0.05

Data are mean values ± standard deviations. Values in each column with different letters (a–c) are significantly different (*p* < 0.05).

**Table 4:** CIELAB color parameters (L*, a*, b*) and total color difference (ΔE) of cassava starch/OPP films across pH values from 1 to 13.

pH	L*	a*	b*	ΔE
1	38.293 ± 0.120	43.293 ± 0.050	41.540 ± 0.295	20.051 ± 0.073
2	39.433 ± 0.170	36.906 ± 0.070	36.463 ± 0.209	17.493 ± 0.161
3	39.326 ± 0.092	33.463 ± 0.032	36.310 ± 0.026	16.935 ± 0.080
4	50.300 ± 0.020	27.020 ± 0.026	35.530 ± 0.026	10.264 ± 0.023
5	47.110 ± 0.010	31.850 ± 0.043	36.460 ± 0.295	10.324 ± 0.142
6	43.703 ± 0.182	34.743 ± 0.081	40.796 ± 0.251	11.560 ± 0.098
7	48.316 ± 0.021	31.820 ± 0.052	38.683 ± 0.015	7.935 ± 0.032
8	45.110 ± 0.010	35.856 ± 0.066	35.903 ± 0.015	12.897 ± 0.038
9	42.270 ± 0.026	37.033 ± 0.041	41.153 ± 0.055	13.569 ± 0.055
10	45.350 ± 0.020	34.273 ± 0.025	38.203 ± 0.025	10.965 ± 0.012
11	40.696 ± 0.025	36.040 ± 0.017	41.190 ± 0.010	14.659 ± 0.008
12	26.856 ± 0.066	34.353 ± 0.041	33.486 ± 0.011	29.548 ± 0.051
13	14.226 ± 0.057	28.170 ± 0.026	17.783 ± 0.020	47.977 ± 0.008

**Table 5:** Microbial growth (APC), pH variation, and total color difference (ΔE) of minced beef meat monitored over 13 days of refrigerated storage using OPP-based pH-sensitive film indicators.

Storage Time (Day)	APC (log CFU/g)	pH	ΔE
0	5.400 ± 0.010 ^c^	6.0430 ± 0.005 ^d^	42.226 ± 0.127 ^a^
3	5.319 ± 0.013 ^d^	6.151 ± 0.007 ^d^	40.845 ± 0.058 ^a^
5	5.348 ± 0.005 ^d^	6.051 ± 0.004 ^d^	44.766 ± 0.029 ^b^
7	5.315 ± 0.015 ^d^	6.083 ± 0.112 ^d^	47.361 ± 0.020 ^c^
9	6.183 ± 0.018 ^b^	6.843 ± 0.035 ^c^	49.655 ± 0.003 ^d^
11	6.213 ± 0.008 ^b^	8.800 ± 0.118 ^b^	58.676 ± 0.021 ^e^
13	6.260 ± 0.010 ^a^	9.570 ± 0.075 ^a^	70.146 ± 0.020 ^f^
*p*-Value	*p* < 0.0001	*p* < 0.0001	*p* < 0.0001

Data are mean values ± standard deviations. Values in each column with different letters (a–f) are significantly different (*p* < 0.05).

## Data Availability

The original contributions presented in the study are included in the article, further inquiries can be directed to the corresponding author.

## Supplementary Materials

The following supporting information can be downloaded at: https://www.mdpi.com/article/10.3390/foods14172974/s1, Figure S1: Representative images of inhibition zones observed in antibacterial tests for cassava starch/onion peel powder (CS/OPP) films.
