# Supplementary material for: pH-Sensitive Cassava Starch/Onion Peel Powder Films as Colorimetric Indicators for Minced Beef Freshness Monitoring
Source: Foods. 2025 Aug 26;14(17):2974. doi: 10.3390/foods14172974 (PMC12428683; doi:10.3390/foods14172974)
Supplement: Supplementary file 1 [file foods-14-02974-s001.zip › foods-3807394-supplementary.pdf]

pH-Sensitive Cassava Starch/Onion Peel Powder Films as Colorimetric Indicators for Minced Beef Freshness Monitoring

Assala Torche [1†], Toufik Chouana [1†*], Ibtissem Sanah [2,3], Fairouz Djeghim [4], Esma Anissa Trad Khodja [5,6], Katiba Mezreb [7], Redouan Elboutachfaiti [8], Cedric Delattre [9], Maria D'Elia [10,11,12] and Luca Rastrelli [10,11,*]

Supplementary Material

Films	*Bacillus cereus*	*Pseudomonas aeruginosa*	Escherichia coli	Staphylococcus aureus
Control	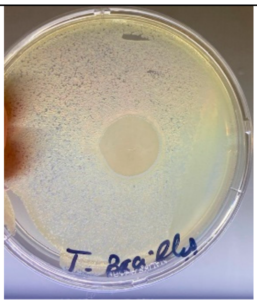	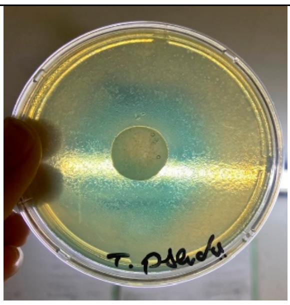	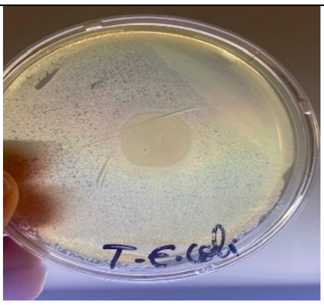	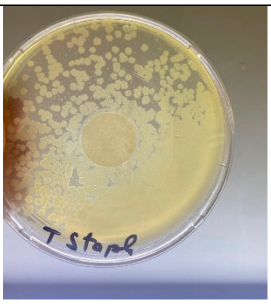
Formula1 (1O)	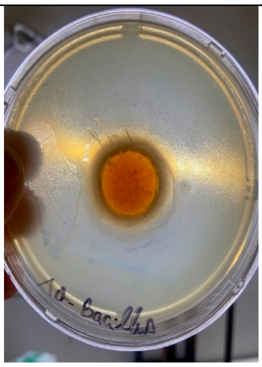	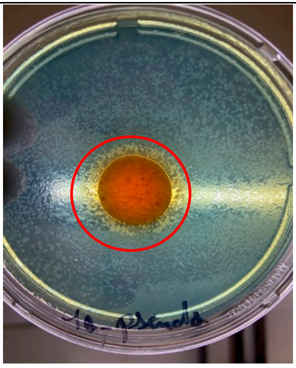	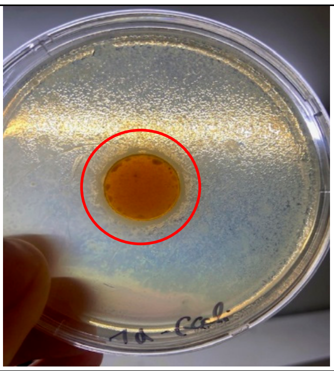	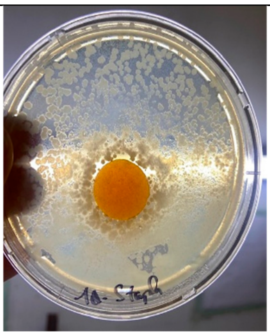
Formula2 (2O)	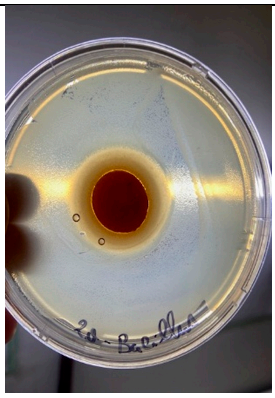	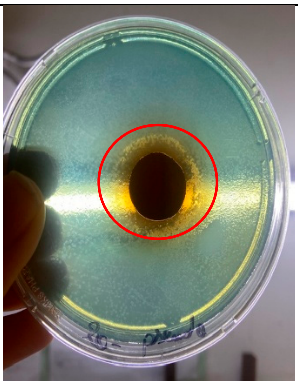	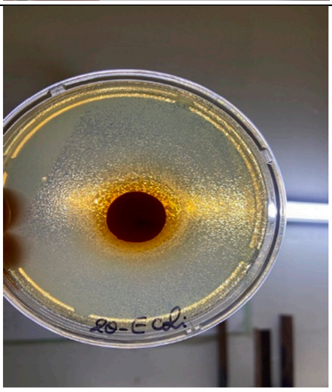	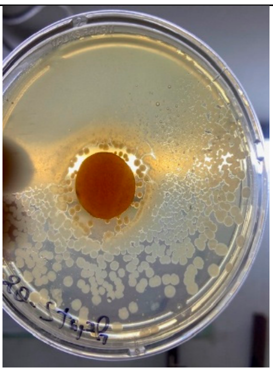

Formula3 (3O)	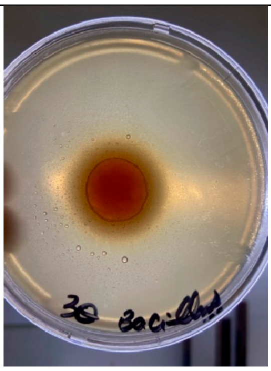	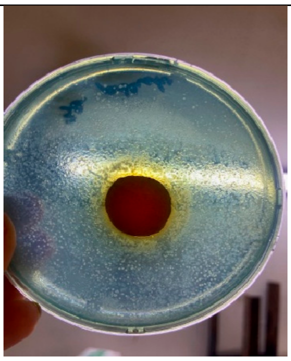	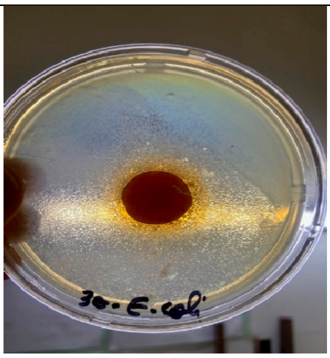	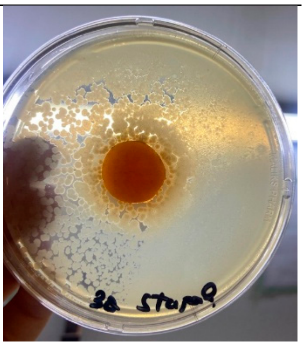

Figure S1. Representative images of inhibition zones observed in antibacterial tests for cassava starch/onion peel powder (CS/OPP) films (formulations 1O, 2O, and 3O) against *Bacillus cereus*, *Pseudomonas aeruginosa*, *Escherichia coli*, and *Staphylococcus aureus*. Images are provided for qualitative illustration; quantitative measurements of inhibition zones are reported in Table X of the main text.
